# Exploring the action mechanism of Gegensan in the treatment of alcoholic liver disease based on network pharmacology and bioinformatics

**DOI:** 10.1097/MD.0000000000038315

**Published:** 2024-06-21

**Authors:** Jiakai Yang, Qianqian Zhuang, Ke Tang, Xinli Liu

**Affiliations:** a Department of Biological Engineering, Qilu University of Technology, Jinan, Shandong Province 250303, China.

**Keywords:** alcoholic liver disease, differential analysis, Gegensan, molecular docking, network pharmacology

## Abstract

Gegensan (GGS) has been reported for the treatment of alcoholic liver disease (ALD), but its therapeutic mechanism is still unclear. This paper aims to determine the therapeutic mechanism and targets of action of GGS on alcoholic liver disease utilizing network pharmacology and bioinformatics. The active ingredients in GGS were screened in the literature and databases, and common targets of ALD were then obtained from public databases to construct the network diagram of traditional Chinese medicine-active ingredient targets. Based on the common targets, Gene Ontology enrichment analysis and Kyoto Encyclopedia of Genes and Genomes (KEGG) analysis were performed to find target enrichment pathways, and the core targets were screened out by combining differential analysis and protein–protein interaction network analysis. Molecular docking was performed to verify the binding effect between the core targets and the corresponding active ingredients. ALD and GGS have 84 common targets, corresponding to 91 active ingredients. After subsequent differential analysis and protein–protein interaction network analysis, 10 core targets were identified. Gene Ontology and KEGG enrichment analyses showed that the main BPs corresponding to the common targets included the response to lipopolysaccharide, inflammatory response, etc. The KEGG pathways involved in the regulation of the common targets included the lipid-atherosclerosis pathway and the alcoholic liver disease pathway, etc. Further molecular docking showed that the core targets CYP1A1, CYP1A2, CXCL8, ADH1C, MMP1, SERPINE1, COL1A1, APOB, MMP1, and their corresponding 4 active ingredients, Naringenin, Kaempferol, Quercetin, and Stigmasterol, have a greater docking potential. The above results suggest that GGS can regulate lipid metabolism and inflammatory response in the ALD process, and alleviate the lipid accumulation and oxidative stress caused by ethanol. This study analyzed the core targets and mechanisms of action of GGS on ALD, which provides certain theoretical support for the further development of GGS in the treatment of ALD, and provides a reference for the subsequent research on the treatment of ALD.

## 1. Introduction

Alcoholic liver disease (ALD) is a chronic liver disease caused by long-term heavy alcohol consumption. The disease starts as an alcoholic fatty liver and later evolves into symptoms such as alcoholic hepatitis, liver fibrosis, and cirrhosis, which in turn causes massive hepatocyte necrosis leading to liver failure.^[1]^ According to the 2017 Global Burden of Disease Study, approximately 27% of patients with cirrhosis and chronic liver disease died from alcohol in 2016.^[2]^ Even in other types of liver disease, alcohol appears as a co-factor, advancing the disease process.^[3]^ According to the “Global Status Report on Alcohol and Health 2018” released by the World Health Organization, total alcohol per capita consumption of adolescents aged 15 years and older increased globally from 5.7 L in 2000 to 6.4 L in 2016.^[4]^ In China, the incidence of alcohol consumption and ALD has also been increasing year by year,^[5,6]^ and an epidemiological survey from Tongzhou District, Beijing, showed that the occurrence of ALD was strongly correlated with hazardous and harmful alcohol consumption, and their population distribution characteristics were generally consistent.^[7]^ ALD cannot be disregarded in light of increasing alcohol consumption, making it all the more crucial to investigate its pathophysiology and discover improved treatment options.

Approximately 90% of ethanol is oxidatively metabolized by the liver, with conversion to acetaldehyde via ethanol dehydrogenase (ADH) being its main metabolic pathway, in addition to the microsomal ethanol oxidation system dominated by cytochromeP450 2E1 (CYP2E1).^[8]^ During this metabolic process, large amounts of reactive oxygen species (ROS) will be generated. Excess ROS buildup causes protein damage, increased fibrillogenesis, and DNA damage, and its interaction with unsaturated fatty acids results in lipid peroxides, which exacerbate hepatocyte damage.^[9,10]^ In addition, hepatocyte injury also results in inflammation and the generation of inflammatory mediators including tumor necrosis factor-α (TNF-α).^[11]^

Gegensan (GGS) is a traditional Chinese medicine formula used to treat the internal toxicity of excessive alcohol consumption (from Rumen Shiqin, Volume 12, by Zhang Zihe, Jin Dynasty). GGS is composed of 5 traditional Chinese herbs, namely, Gegen (radix puerariae, GG), Gehua (puerariae flos, GH), Gancao (licorice, GC), Sharen (amomum, SR), and Guanzhong (fortunes bossfern rhizome, GZ). Taotao Zhou et al found that GGS reduced TNF-α expression and was effective in ALD.^[12]^ Senqin Liu et al showed that GGS reduced serum alanine aminotransferase, aspartate aminotransferase and total bilirubin levels in rats and alleviated liver injury caused by ALD.^[13]^ Weiyi Tian et al found that GGS substantially reduced the expression level of Caspase3 and alleviated liver injury in acute alcoholism.^[14]^ In addition, GGS is also effective against colorectal cancer and colon adenocarcinoma.^[15,16]^ However, no study has yet outlined the molecular basis of how GGS works to affect ALD, and understanding this basis will be crucial for future GGS applications.

Chinese medicine formulas have complex components and numerous corresponding targets, which are difficult to analyze at the molecular level. The concept of network pharmacology was first proposed by Hopkins et al who believed that it would be a new paradigm for drug research.^[17,18]^ Network pharmacology can visualize complex herb-compound-target-disease relationships through computer-simulated network models, which helps pharmacological research in Chinese medicine.^[19]^ In this study, network pharmacology methods were used to obtain the corresponding targets of the compounds contained in different components of GGS and the targets corresponding to ALD by querying various databases. Then, protein interaction network, Gene Ontology (GO), Kyoto Encyclopedia of Genes and Genomes (KEGG), and Gene Expression Omnibus (GEO) datasets were used to filter comparison to discover the core targets once the common targets were established. Finally, a combination of molecular docking was used for validation. In this way, this study provide theoretical support for the subsequent GGS treatment of ALD.

## 2. Materials and methods

### 2.1. Active ingredient and target screening of GGS

The herbal compounds and the corresponding targets in GGS were collected from the the Traditional Chinese Medicine Systems Pharmacology Database (TCMSP, https://tcmsp-e.com), Bioinformatics Analysis Tool for Molecular mechANism of Traditional Chinese Medicine (BATMAN-TCM, http://bionet.ncpsb.org.cn/batman-tcm/index.php/), Traditional Chinese Medicines Integrated Database (TCMID, https://bidd.group/TCMID/index.html) and other databases.^[20–22]^ Eligible compounds were screened based on oral availability (oral availability ≥ 30%) and drug-like (drug-like ≥ 0.18), after which individual compounds were corresponded to their targets and compounds without targets were excluded. A total of 103 compounds with 252 corresponding targets were obtained.

### 2.2. ALD target screening

The keyword “alcoholic liver disease” was used to obtain the relevant targets in DisGeNET (https://www.disgenet.org/) and Genecard (www.genecards.org).^[23–25]^ One hundred ninety-three relevant targets were obtained in DisGeNET, and 431 relevant targets were obtained in Genecard based on the correlation score of ≥30. After merging and de-duplication, a total of 513 disease targets were obtained. The Venn diagram of compounds targets and ALD targets were drawn using the R package venn.

### 2.3. Construction and optimization of herbal-compound-common target network

Because of the large number of compounds and targets in the Chinese herbal formulae, it is difficult to obtain the required information for the network diagram, so only the common targets of ALD and GGS and their corresponding compounds were chosen for this network diagram. Cytoscape v3.9.0^[26]^ was used to draw the TCM-compound-target network diagram, and the transparency, size and color of each node were adjusted according to the degree of degree.

### 2.4. Protein–protein interaction (PPI) network mapping and optimization

Interaction data for common targets were obtained in the STRING database (https://cn.string-db.org/)^[27]^ and isolated targets were removed. The nodes were adjusted in Cytoscape v3.9.0 according to the degree value size. In addition, by calculating the values of degree, closeness and betweenness for each node, targets larger than the mean of each value were selected and labeled. In addition, all nodes were grouped into 3 clusters by K-means clustering to better identify the patterns. The nodes of the clustered targets were optimally adjusted in Cytoscape v3.9.0 according to the values of degree, where the thickness of the line between targets was adjusted according to the combined score.

### 2.5. Core target screening

#### 2.5.1. GEO datasets gene expression differential analysis

The GEO datasets (https://www.ncbi.nlm.nih.gov/geo/)^[28]^ contain clinical data for various diseases and can obtain mRNA expression information for various diseases. To further screen the core targets of GGS for ALD, 2 gene chips, GSE28619 and GSE100901, were screened using “alcoholic liver disease” as a keyword. The former contains 7 normal groups as well as 15 diseased groups, while the latter contains 4 normal groups and 4 diseased groups. The latter were normalized due to the large differences and a box plot of expression values before and after treatment was plotted (Fig. S1, Supplemental Digital Content, http://links.lww.com/MD/M826). After the data were processed, a heat map of gene expression differences (showing only the top 100 genes in terms of difference size) and a volcano map were plotted by R software.

#### 2.5.2. GO and KEGG analysis

The DAVID database (https://david.ncifcrf.gov/)^[29,30]^ is a commonly used website for GO as well as KEGG, and the data have been recently updated and are more reliable. GO analysis includes 3 aspects: biological process (BP), molecular function (MF) and cellular composition. GO analysis allows finding GO taxonomic entries enriched by common genes and understanding the gene functions they act on, while KEGG analysis makes it easier to discover their action mechanisms. Data obtained from GO and KEGG analyses were plotted using R software and Origin 2022 software.

### 2.6. Molecular docking

The macromolecular structure files were obtained from AlphaFold database (https://alphafold.ebi.ac.uk/),^[31,32]^ PDBe-KB database (https://www.ebi.ac.uk/pdbe/pdbe-kb/),^[33]^ and saved after removing water molecules and deleting ligands. The structures of the TCM compounds corresponding to the core targets were obtained from the Pubchem database (https://pubchem.ncbi.nlm.nih.gov/)^[34]^ and docked in Autodock v1.5.6.^[35]^ The docking results were analyzed on the PLIP website (https://plip-tool.biotec.tu-dresden.de/plip-web/plip/index)^[36]^ as a way to obtain interactions between ligands and proteins other than hydrogen bonds. The final images were optimized by pymol software.^[37]^ The docking results were mapped by Ligplot + v2.2^[38]^ for protein-ligand 2D interactions.

The search for docking was limited to the active center region, and the selection of the best docking results followed the following requirements: it must be in the region of the reported active center; hydrogen bonding must be present; and for the binding conformation in the active center, the one with the lowest binding energy was selected. If no relevant active center was reported, the active site was predicted through the DEEPSITE website (https://playmolecule.com/deepsite/).^[39]^

After the docking was completed, a heat map labeled with the size of the docking energy was drawn through the Microsun website (http://www.bioinformatics.com.cn/).^[40]^

### 2.7. Optimization of KEGG pathway diagram and other images

The R package Pathview was used to label the core targets obtained by screening on the pathway they were on. The up and down adjustment of the targets was distinguished by color change. Metabolic pathway maps etc were drawn via Adobe Illustrator 2022 and some of the material in the figure was obtained from the smart servier website (https://smart.servier.com) and the reactome database (https://reactome.org/).^[41]^

## 3. Results

### 3.1. Construction of herbal-compound-target network

**Figure 1.**
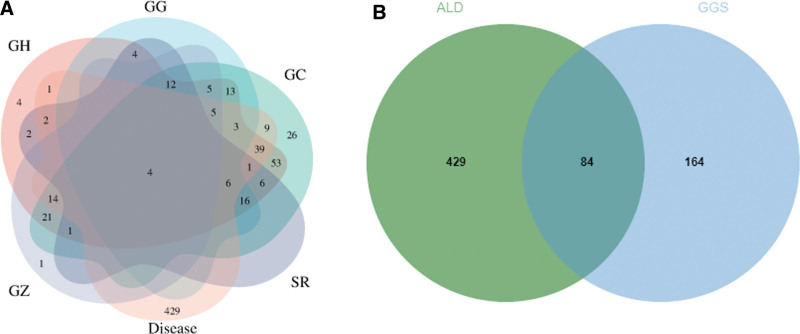
Screening of common ALD-GGS targets. (A) Venn diagram of each component of GGS and ALD targets (disease refers to ALD by proxy). (B) Venn diagram of the corresponding targets of GGS and all targets of ALD.

One hundred twenty compounds were recorded in GGS. GG, GH, GC, GZ, and SR in GGS correspond to 68, 218, 234, 54, and 63 targets, respectively, for a total of 252 different targets. One hundred ninety-four relevant targets were obtained from the DisGeNET website after removing duplicates using “alcoholic liver disease” or “alcoholic fatty liver disease” as keywords. Using the same keywords, 381 targets were obtained by screening the target proteins with correlation scores ≥30 through the Genecard website. Venn diagram of each component of GGS and ALD targets showed that GH, GC, and ALD had more common targets. Venn diagrams of all ALD targets and GGS showed that ALD and GGS had 84 common targets (Fig. 1).

**Figure 2.**
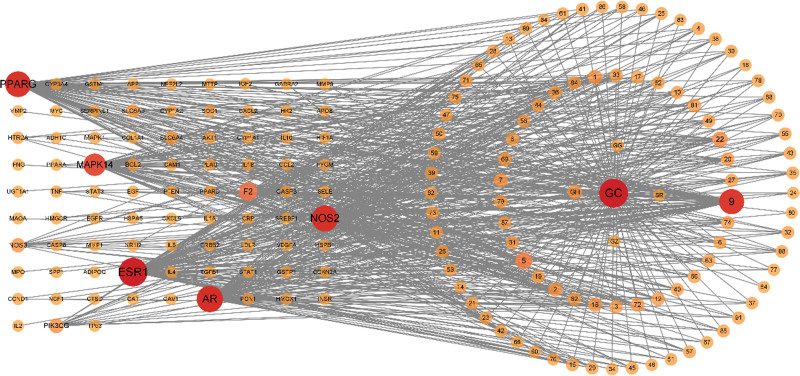
GGS herbal-compound-target network diagram. The rectangular dot matrix on the left is the target, the 2 circles outside the concentric circles on the right are the compound numbers, and the inner is the herbal component, the node color and size become larger with the degree value.

After excluding the disease-independent targets corresponding to GGS, the herbal medicine, herbal compounds, and common targets of herbal medicine and disease were imported into Cytoscape 3.9.0 to construct the herbal medicine-compound-target network. Without the interference of redundant disease-independent targets, it can better reflect the correspondence between each target and compound (Fig. 2). The network showed that GC corresponded to the most compounds with a common target among the 5 medicines, and quercetin (degree:9) corresponded to the most targets among the single compounds.

### 3.2. GGS-ALD key target analysis

#### 3.2.1. Construction of common target PPI network

**Figure 3.**
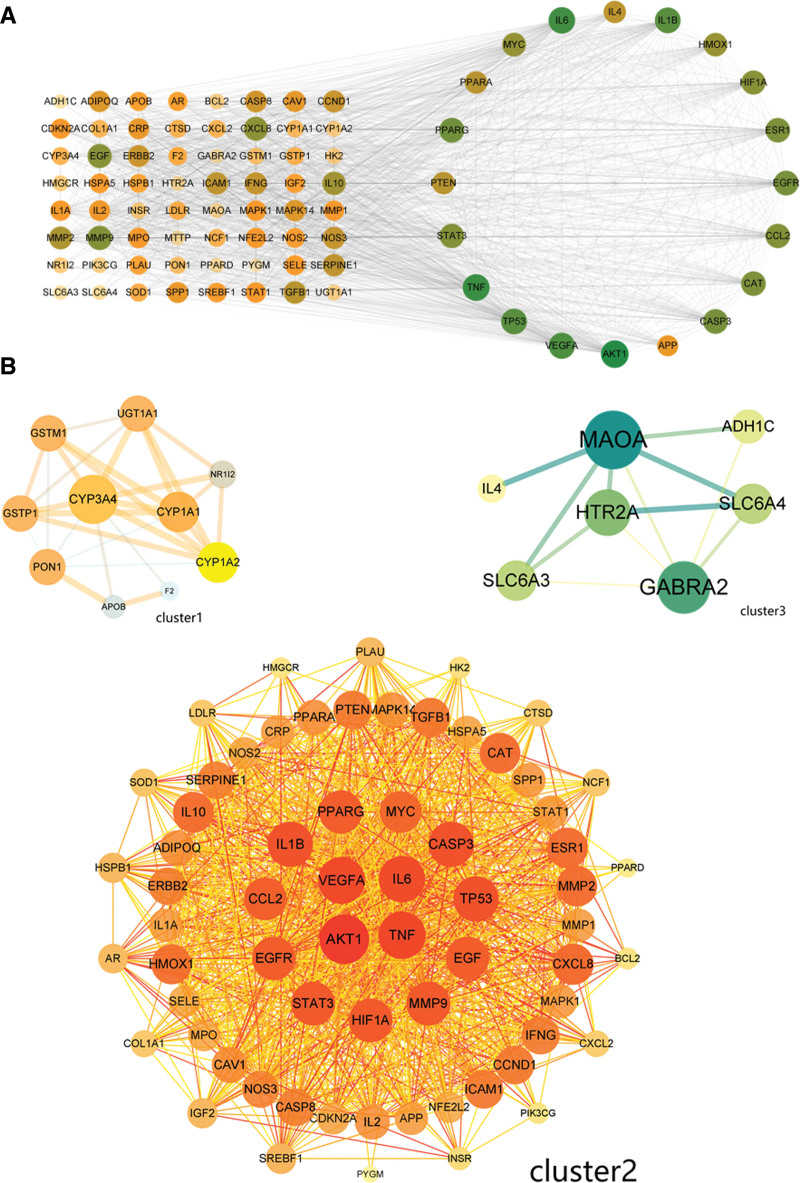
PPI network analysis of the corresponding target of GGS and the common target of ALD target. (A) Common PPI analysis of all network nodes with 3 values of degree, closeness, and betweenness greater than the threshold on the right. Node color, size, and degree values are proportional to each other. (B) Clustering analysis network of nodes based on K-means clustering. Node color, size, and degree value are proportional to each other, and the color and thickness of the lines are proportional to the correlation.

Protein PPI networks were constructed to further reveal the mechanism of GGS for ALD treatment. The 84 common targets were input into String website to construct their PPI networks, and the data were imported into Cytoscape 3.9.0 for further analysis. The mean values of degree, closeness, and betweenness of each node were 35.21, 0.0076, and 52.26, respectively, which were calculated by the Centiscape 2.2 plug-in. Then, 3 nodes whose values were all greater than the mean values were initially selected and set as a circular distribution on the right side and the others as a matrix distribution on the left side to obtain 20 targets with large associations with other proteins (Fig. 3A). In addition, the original PPI network was clustered into 3 clusters by k-means clustering, including 10, 66, and 8 targets, respectively (Fig. 3B).

#### 3.2.2. Results of variance analysis based on GEO datasets

There are more targets obtained by the above steps, and it is still biased to define the core targets by only relying on the numerical calculation of network nodes. Therefore, additional screening of the core targets is still required. We obtained 2 ALD-associated gene chips, GSE28619 and GSE100901, from the GEO datasets, and normalized the data for GSE100901 because of excessive variability. The GSE28619 database contained 7 normal samples and 15 ALD samples. The GSE100901 database contained 4 normal samples and 4 ALD samples. The 2 databases were differentially analyzed using limma, heat map, and ggplot2 packages in R software, and genetic differential heat map and volcano map were plotted for the 2 databases, respectively.

**Figure 4.**
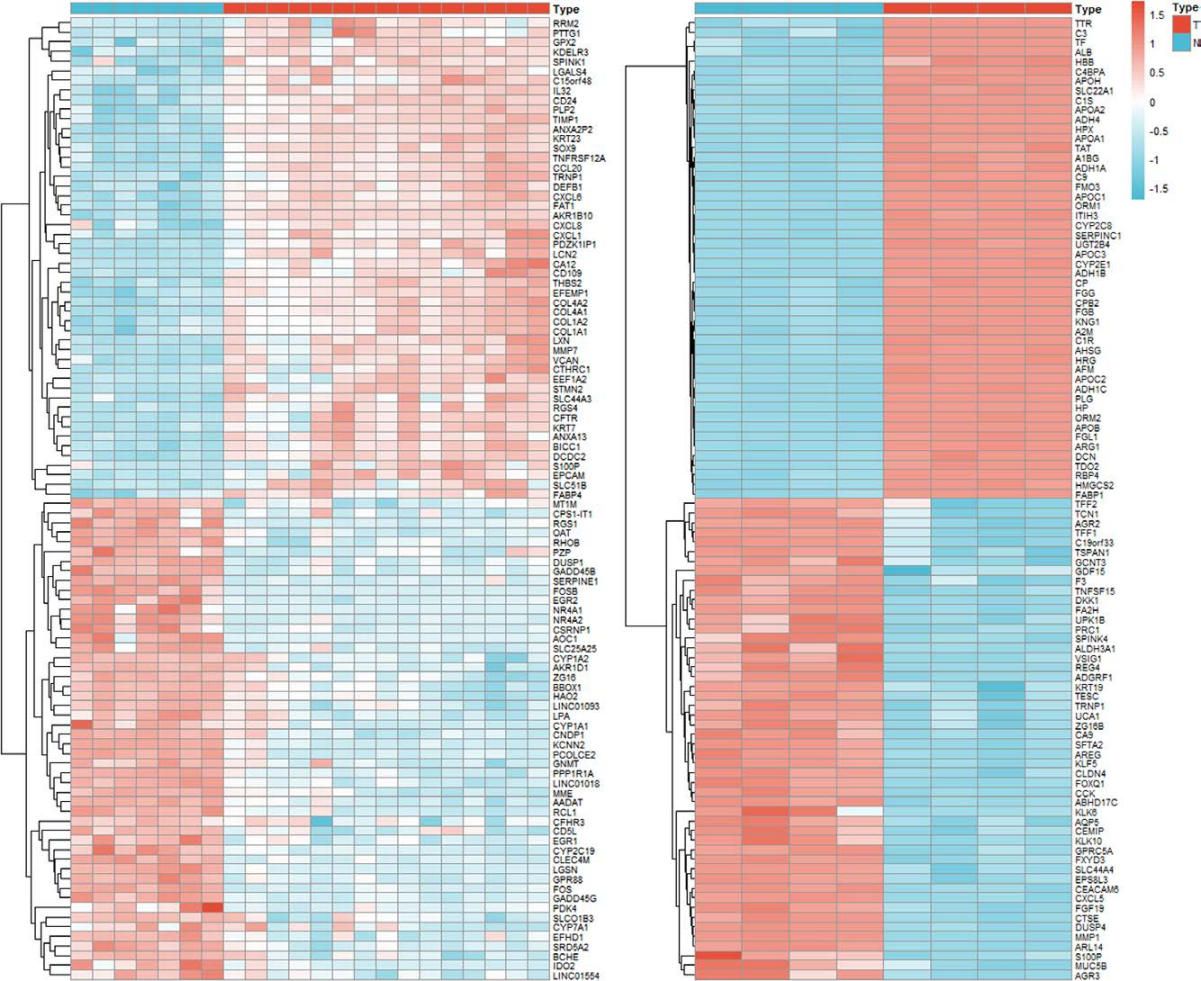
Heat map of the difference in gene expression between GSE28619 and GSE100901. GSE28619 on the left and GSE100901 on the right, blue is down-regulated and red is up-regulated, the color shades are proportional to the absolute value of expression.

**Figure 5.**
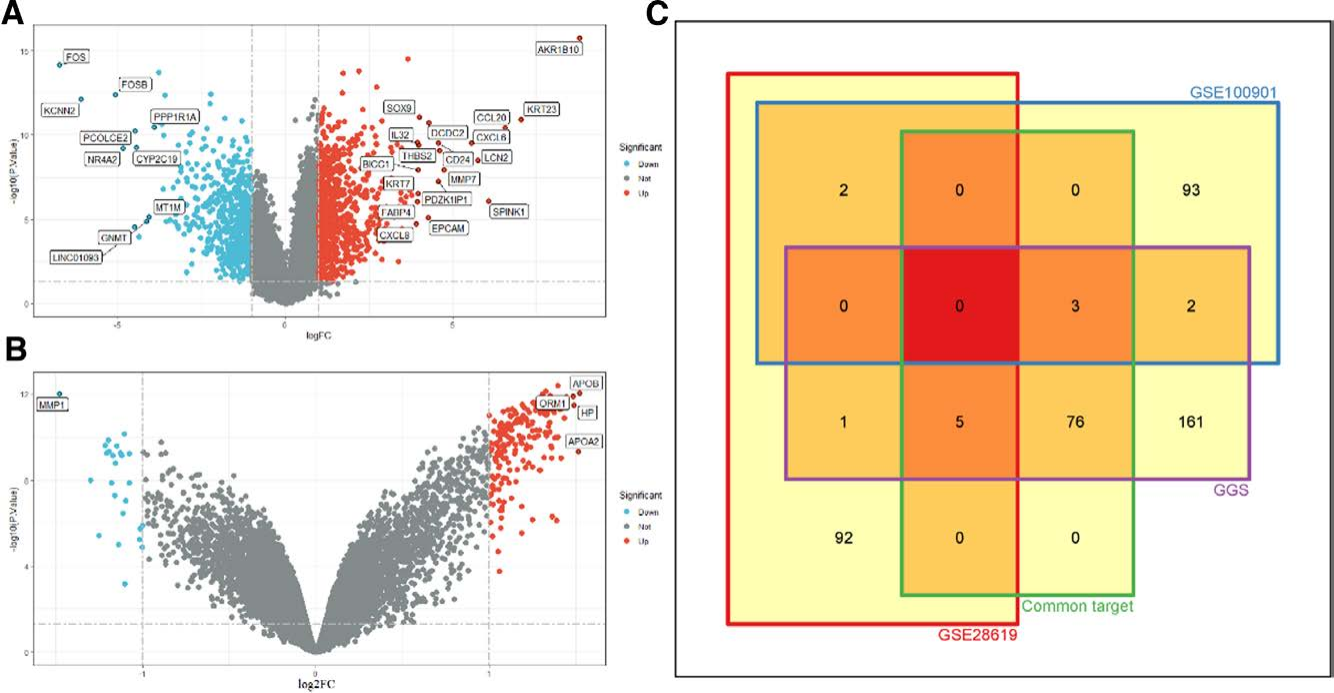
Gene expression difference volcano plot and Venn diagram for GES28619 and GSE100901. (A) GSE28619 gene expression difference volcano plot, genes with logFC > 3.8 and ‐log10(P.Value) > 4 were considered as significant expression differences and labeled. (B) GSE100901 gene expression difference volcano plot, genes with log2FC > 1.45, ‐log10(P.Value) > 4 were considered as expression difference significant and labeled. (C) Venn diagram of GES28619, GSE100901 heat map genes, GGS–ALD common target, and the corresponding target of GGS.

The top 100 genes ranked by the magnitude of the difference (i.e., absolute value of logFC or log_2_FC) are shown in the heat map (Fig. 4). CYP1A2, CYP1A1, SERPINE1, CXCL8, and COL1A1 in the GSE28619 database overlap with the common target (Fig. 4A), and MMP1, ADH1C, and APOB in the GSE100901 database overlap with the common target (Fig. 4B). In addition, the non-common target targets belonging to GGS, FOS, F3, and CLDN4, also showed significant expression differences. Based on these genes, they were linked to the corresponding compounds from the network pharmacology images, and the corresponding compounds included Kaempferol, Quercetin, Stigmasterol, etc (Table 1). In the volcano map, the overlapping genes are shown to be differentially expressed at higher levels than the others in APOB, CXCL8, FOS, and MMP1, etc (Fig. 5A and B).

**Table 1:** Comparison table of target sites with corresponding gene sets and compounds.

Gene name	Protein name	Gene set	Corresponding compound
CYP1A2	Cytochrome P450 1A1	GSE28619 common target GGS	Kaempferol Quercetin
SERPINE1	Plasminogen activator inhibitor 1	GSE28620 common target GGS	Quercetin
CYP1A1	Cytochrome P450 1A2	GSE28621 common target GGS	Kaempferol Quercetin
COL1A1	Collagen alpha-1(I) chain	GSE28622 common target GGS	Quercetin
CXCL8	Interleukin-8	GSE28623 common target GGS	Quercetin
FOS	Proto-oncogene c-Fos	GSE28619 GGS	Kaempferol beta-sitosterol
MMP1	Matrix metalloproteinase-1	GSE100901 common target GGS	Kaempferol Quercetin
ADH1C	Alcohol dehydrogenase 1c	GSE100901 common target GGS	Stigmasterol
F3	Coagulation factor iii	GSE100901 GGS	Quercetin
CLDN4	Claudin-4	GSE100901 GGS	Quercetin
APOB	Apolipoprotein B	GSE100901 GGS	Naringenin

### 3.3. Results of GO and KEGG analyses

The key genes obtained in the differential analysis were distributed in different clusters (Fig. 5C), and the linkage could not be visualized yet, so GO and KEGG analyses were needed to further explore the action mechanism of GGS on ALD.

**Figure 6.**
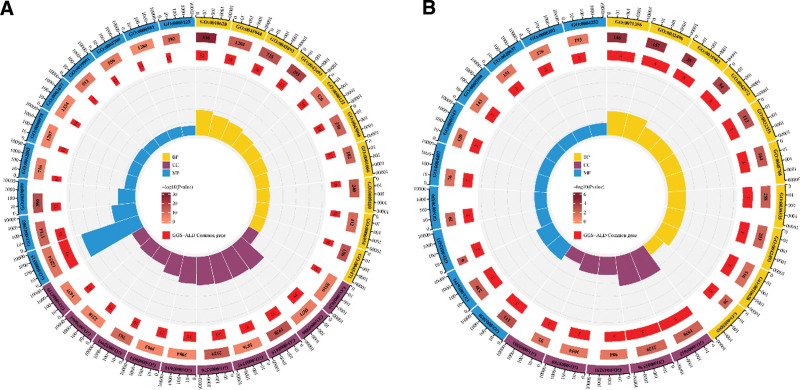
Circular plot of GO enrichment results. The values from outside to inside are, in order: classification, the total number of genes for the gene entry, the number of genes enriched to the original gene set. The height of the bar graph is all genes in the original gene set/number of genes enriched to the original gene set.

Based on 84 overlapping targets of GGS-ALD to perform GO analysis, BP included 31 entries, cellular composition included 20 entries, and MF included 15 entries, the top 10 gene enrichment entries were selected to plot a circular graph of GO enrichment results (Fig. 6A). The main BPs include positive regulation of gene expression (GO:0010628), response of DNA template to drugs (GO:0045893), negative regulation of apoptotic process (GO:0043066), response to stimulation by exbiotics (GO:0009410), inflammatory response (GO:0006954), response to ethanol (GO: 0045471), etc. These BPs are either directly related to ethanol metabolism or to the inflammation caused by ethanol-induced damage to hepatocytes (Fig. 6A). The main MFs include protein binding (GO:0002020), enzyme binding (GO:0019899), etc.

GO analysis of the core targets showed that the main BPs were concentrated in the cellular response to TNF (GO:0071356), response to lipopolysaccharide (GO:0032496), response to braking stress (GO:0035902), retinol metabolic process (GO:0042572), and response to drugs (GO:0042493) (Fig. 6B). The main MFs are concentrated in the entry of oxidoreductase activity (GO:0016491) in addition to protein binding.

**Figure 7.**
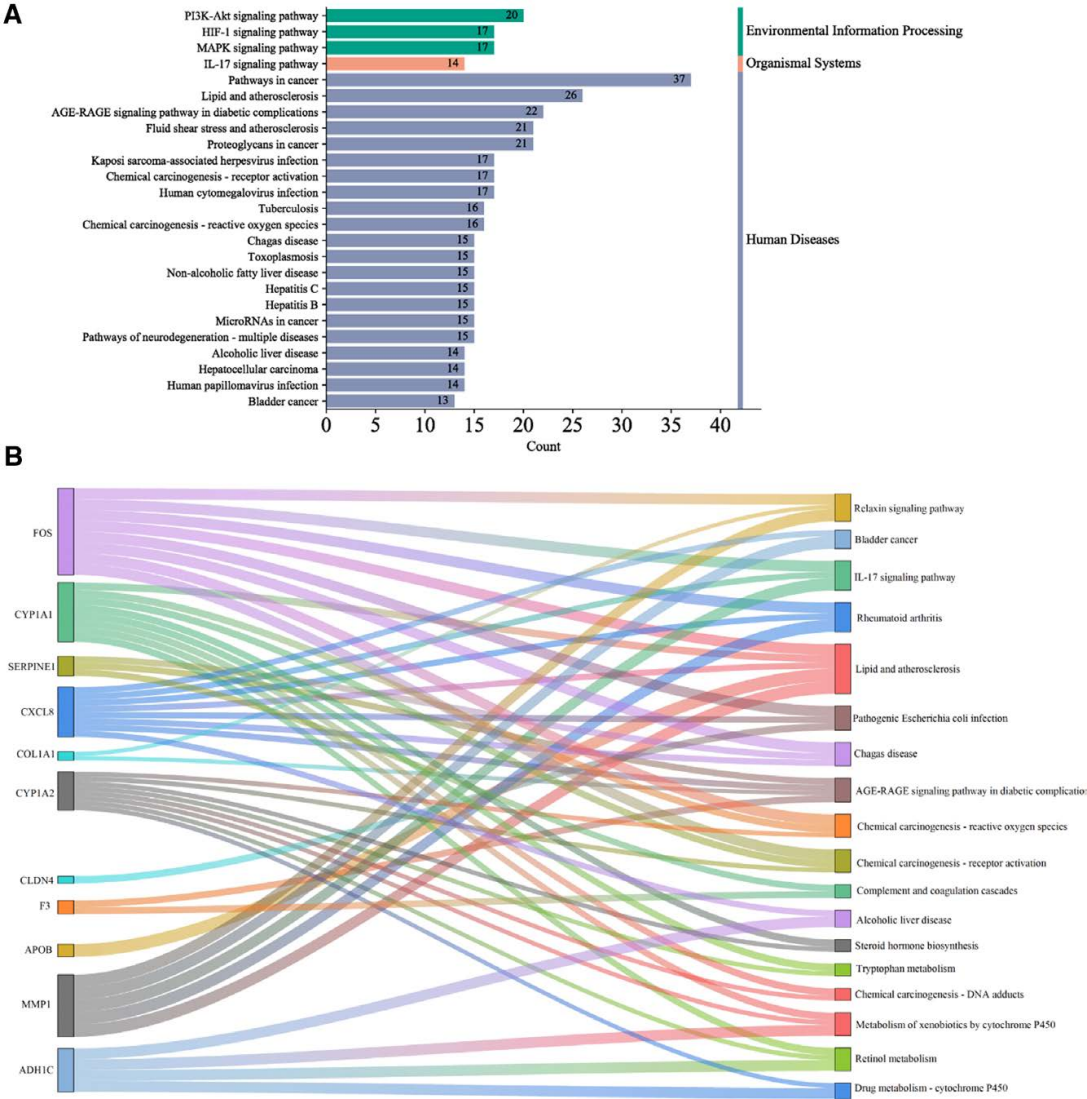
KEGG analysis. (A) Classification diagram of Pathway enrichment results for 84 common targets of GGS–ALD. (B) Sankey diagram of the core targets corresponding to the pathway. The targets derived from GSE28619 and GSE100901 are each divided into 2 parts and sorted according to the logFC value, and the thickness of the target-pathway linkage is proportional to the logFC value.

**Figure 8.**
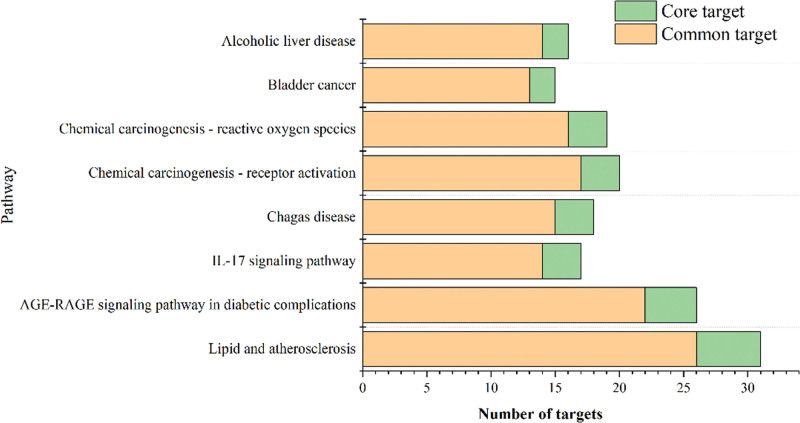
Stacked plot of enrichment results for the common pathway.

KEGG analysis of 84 overlapping targets showed that there were 65 pathways with ≥10 enriched genes, and the top 25 enriched genes included cancer pathway, lipid and atherosclerosis, MAPK signaling pathway, IL-17 signaling pathway, and liver cancer pathway, in addition to the directly related pathway of ALD (Fig. 7A). The core targets correspond to 18 pathways, CXCL8, CY1A2, FOS, and other targets correspond to multiple pathways, and the most corresponding pathway is lipid and atherosclerosis pathway (Fig. 7B). Among the overlapping pathways, the lipid and atherosclerosis pathways also have the highest number of genes, while AGE-RAGE-related pathways, inflammation pathways and chemical carcinogenesis-ROS pathways also have more genes enriched (Fig. 8).

### 3.4. Molecular docking of core targets

Through the above analysis, CYP1A1, CYP1A2, CXCL8, ADH1C, MMP1, SERPINE1, and COL1A1 were identified as core targets. APOB and MMP1 were also added as core targets since they are both targets with large differential expression in GSE100901 and share the same metabolic pathway of lipids and atherosclerosis. APOB corresponds to Naringenin, MMP1, CYP1A1, and CYP1A2 correspond to both Kaempferol and Quercetin, CXCL8, COL1A1, and SERPINE1 correspond to Quercetin only, and ADH1C corresponds to Stigmasterol (Table 1).

**Figure 9.**
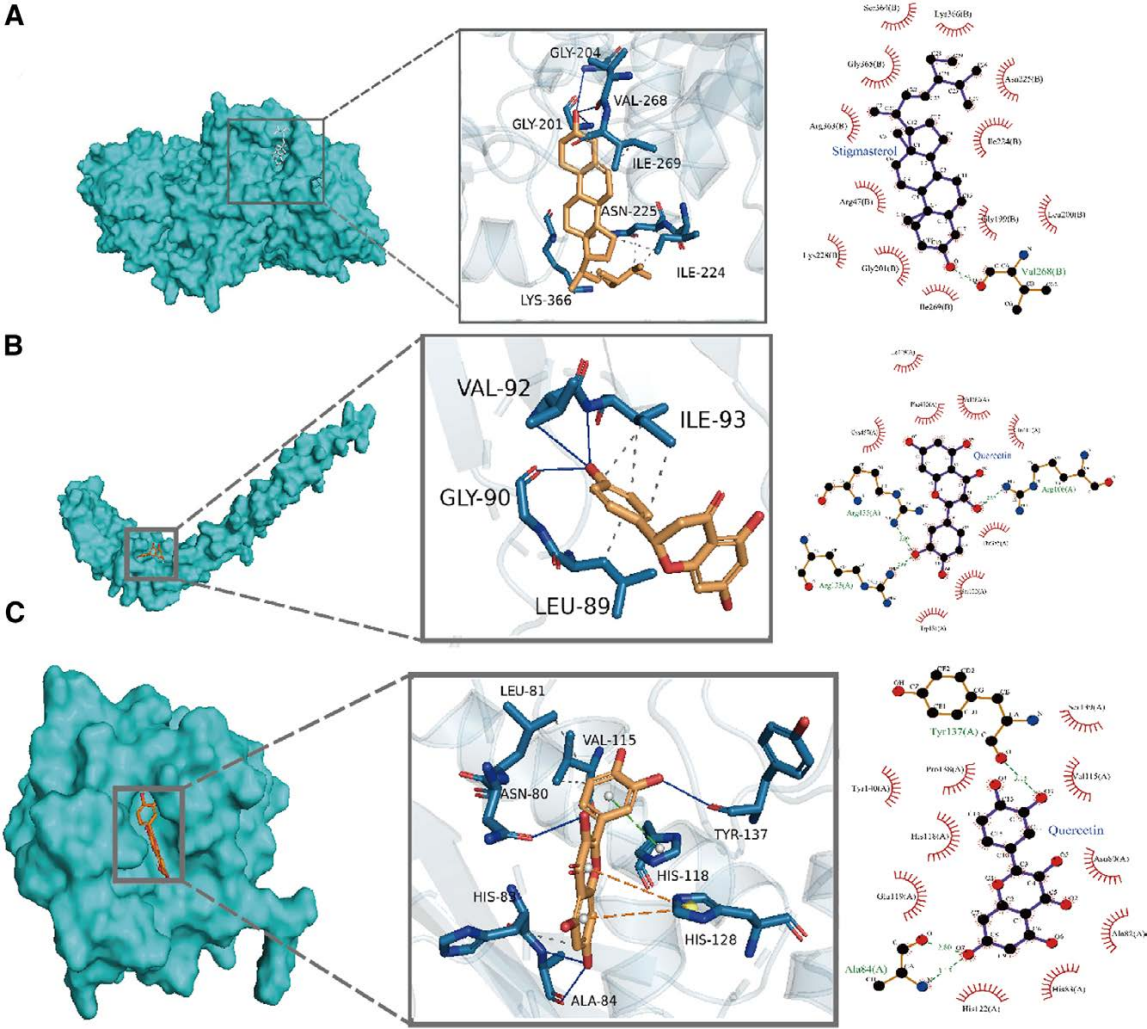
Binding conformation of key targets and compounds molecularly docked. (A) ADH1C and Stigmasterol. (B) APOB and Naringenin. (C and D) MMP1 and Quercetin.

**Figure 10.**
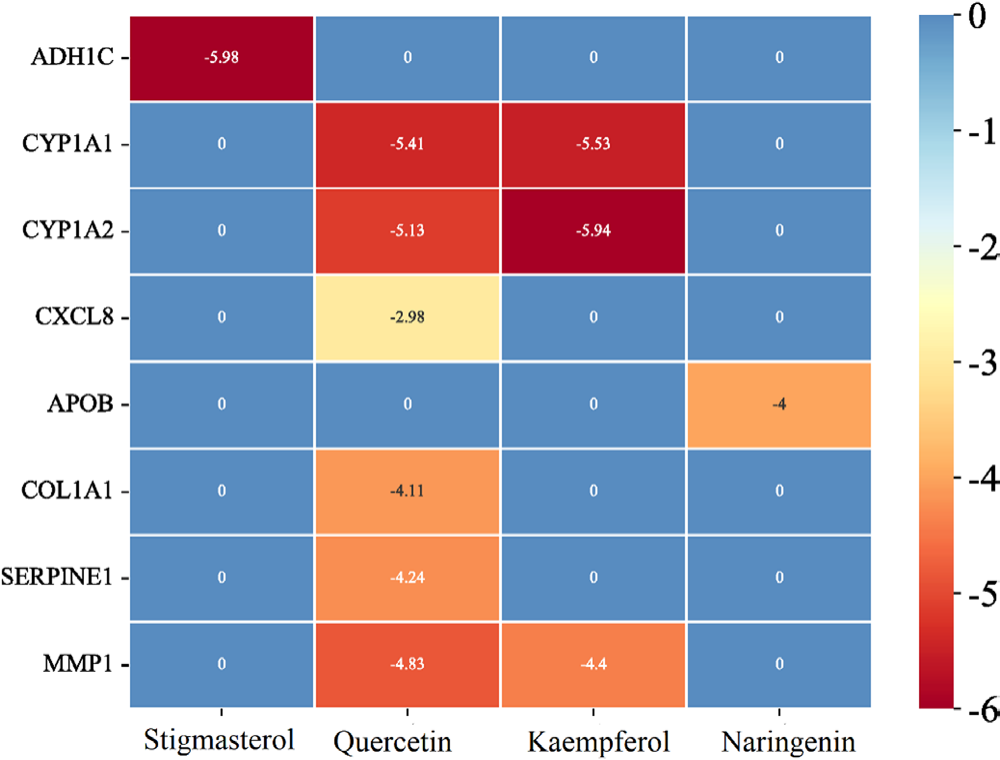
Docking energy heat map of target proteins and corresponding compounds. Non-corresponding relationships are marked with the number 0.

A preliminary analysis of the docking effect of the core target and the compound was performed based on molecular docking. ADH1C has 3 possible amino acid sites for hydrogen bond formation at the active center and Stigmasterol. The result of its hydrogen bond formation with the amino acid Val268, which has the shortest hydrogen bond distance, is shown in the 2D diagram of protein–ligand interactions, and its surrounding hydrophobic amino acids provide a better hydrophobic microenvironment (Fig. 9A). Docking of APOB and Naringenin resulted in 3 possible hydrogen bond binding sites, including Ily93, Gly90 (Fig. 9B). Quercetin corresponded to COL1A1, CXCL8, CYP1A1, CYP1A2, MMP1, SERPINE1, among which CYP1A2, SERPINE1 docking resulted in the largest number of hydrogen bonds, 5 in total (Fig. S2, Supplemental Digital Content, http://links.lww.com/MD/M827). Kaempferol has 2 hydrogen bonding sites with both CYP1A1 and CYP1A2, while the docking with MMP1 has 3 hydrogen bonds, along with other acting forces such as π-stacking (Fig. 9C). Among all docking results, ADH1C showed the lowest binding energy with Stigmasterol. In addition, the lowest binding energy with quercetin was CYP1A1, the lowest binding energy with Kaempferol was CYP1A2, and the lowest binding energy compound corresponding to MMP1 was quercetin (Fig. 10).

## 4. Discussion

**Figure 11.**
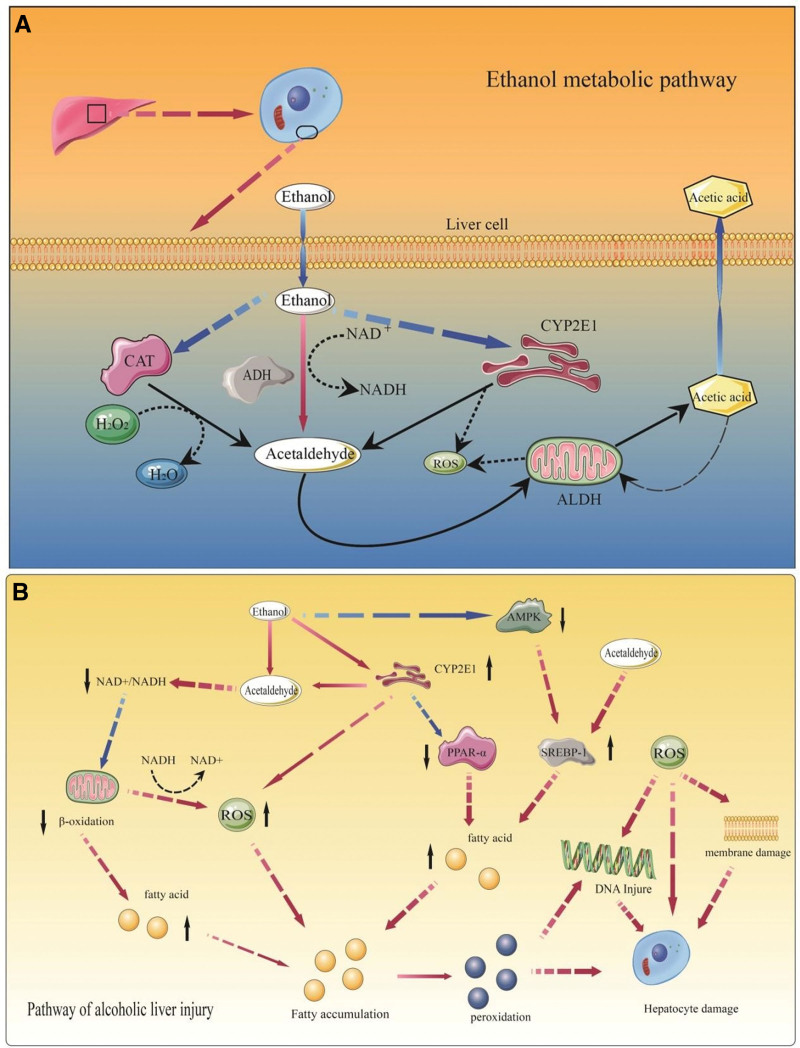
The process of alcoholic liver injury formation. (A) Ethanol metabolism pathway. (B) Alcoholic liver injury pathway.

The primary pathway of ethanol metabolism in the liver is through the conversion of ADH to acetaldehyde, while the secondary pathway involves CYP2E1 in the endoplasmic reticulum’s microsomal oxidative system, which also converts ethanol to acetaldehyde but with the generation of ROS.^[8]^ Alternatively, hydrogen peroxide also converts ethanol to acetaldehyde, and all the acetaldehyde produced is converted to acetic acid by aldehyde dehydrogenase. Acetic acid is less metabolized in the liver and most of it enters the peripheral circulation and flows with blood transport to various tissues (Fig. 11A).

The pathogenesis of ALD is complex and includes multiple factors such as steatosis, oxidative stress, and immune damage. ALD first manifests as a fatty liver due to fat accumulation, which then evolves into inflammation and fibrosis. The conversion of ethanol to acetaldehyde by ADH consumes large amounts of NAD+, which affects the NAD+/NADH ratio, allowing the fatty acid oxidation step to be inhibited, resulting in fat accumulation. The reoxidation of NADH by mitochondria will in turn be accompanied by the generation of ROS.^[42–44]^ Acetaldehyde itself can form covalent compounds with proteins, fats, and DNA, leading to structural changes in proteins and DNA damage. In addition, the family of peroxisome proliferator-activated receptors (PPARs) plays a role in the production of fatty liver. PPARα can regulate the transcription of genes related to fatty acid esterification and production and is involved in regulating the fatty acid oxidation step. It has been demonstrated that PPARα deficiency will lead to fat accumulation and that acetaldehyde can interfere with the transcriptional activity of PPARα.^[45]^ In addition, upregulation of oxidative stress produced by ethanol through CYP2E1 metabolism also inhibits PPARα activity. Sterol regulatory element-binding proteins (SREBPs) are closely associated with PPARs and are involved in fatty acid and triglyceride synthesis. Acetaldehyde can increase the level of SREBP-1 and thus induce hepatic fat accumulation.^[46]^ AMP-activated protein kinase (AMPK) can have a regulatory effect on SREBP-1. AMPK phosphorylation regulates enzymes involved in lipid metabolism, and AMPK expression is downregulated in chronically alcohol-exposed mice.^[47]^ Fat accumulation ultimately leads to the development of fatty liver. CYP2E1 expression is increased under chronic ethanol exposure, resulting in increased ROS production.^[48,49]^ ROS produced in ethanol metabolism will promote lipid peroxidation and the production of protein adducts, which will disrupt cell membranes. In addition, lipid peroxidation can further activate adaptive immunity by combining with acetaldehyde and proteins into larger adducts, increasing circulating levels of pro-cellular inflammatory factors, which leads to alcohol-derived pro-inflammatory effects^[9]^ and further aggravates alcoholic liver injury (Fig. 11B).

GGS has a therapeutic effect on ALD,^[12–14]^ but its molecular mechanism is not yet clear. In the drug-compound-target network, there are 84 common ALD-GGS targets and 91 compounds related to the common targets, among which licorice corresponds to the most compounds and quercetin corresponds to the most targets (Fig. 2). The top 3 degree values of compounds also include Kaempferol (name: 5), Naringenin (name: 22). Studies have shown that quercetin has an ameliorative effect on high-fat diet-induced fatty liver in mice and can modulate the gut microbial community that is disrupted by high-fat diet.^[50]^ In an in vitro cellular model of ALD, quercetin reduces the level of pro-cellular inflammatory factors and ameliorates the cellular damage caused by ROS.^[51]^ Ethanol increases intestinal permeability, causing lipopolysaccharides derived from the intestine to enter the circulation. Kaempferol alleviates the resulting cellular inflammatory response and stabilizes endothelial barrier damage.^[52]^ Naringenin, on the other hand, reduces lipid accumulation by increasing AMPK activity and can inhibit inflammation and improve liver fibrosis.^[53,54]^ This suggests that GGS is influencing the ALD development process in several ways.

Differential analysis of the GSE28619 gene chip revealed that the expression of common ALD-GGS targets CYP1A2, CYP1A1, SERPINE1, CXCL8, COL1A1 varied significantly between normal and ALD samples, with CXCL8 showing the greatest variation. Furthermore, MMP1, APOB, and ADH1C were selected as core targets in GSE100901. These targets are strongly correlated with inflammatory response, ethanol metabolism, and lipid metabolism. Analysis of the bioprocess enrichment of common ALD–GGS targets showed that most of the target genes were enriched in the apoptotic process regulation, inflammatory response, and ethanol response entries, a result that is consistent with the results of the differential analysis. KEGG analysis showed that both common and core targets were enriched in lipid and atherosclerotic pathways. In the lipid and atherosclerosis pathway map, APOB in the core target is involved in early lipid droplet formation, and APOB gene expression is upregulated in ALD patients as shown in the GSE100901 database. The GSE100901 database shows that MMP1 is downregulated and linked to liver fibrosis. This downregulation would encourage the replacement of the extracellular matrix by the sclerotic matrix,^[55]^ further leading to autophagy. In addition to this, more targets are enriched in the oxidative stress and inflammatory response link induced by ROS generated by AGE-RAGE signaling pathway, corroborating that the therapeutic effect of GGS on ALD is reflected in this link (Fig. S3, Supplemental Digital Content, http://links.lww.com/MD/M828). As mentioned earlier, inflammatory response due to oxidative stress is one of the important causes of alcoholic liver injury. In the pathway map of ALD, it was shown that the targets of GGS for ALD are involved in regulating PPARA and other genes related to lipid metabolism in addition to downstream inflammation-related targets (Fig. S4, Supplemental Digital Content, http://links.lww.com/MD/M829). This result corresponds to the results of KEGG analysis.

The core targets CYP1A1, CYP1A2, CXCL8, ADH1C, MMP1, SERPINE1, COL1A1, APOB, and MMP1 were molecularly docked with the corresponding compounds Naringenin, Kaempferol, Quercetin, and Stigmasterol to evaluate whether they could produce effects. The docking results showed that the docking binding energy ranged from ‐2.98 to ‐5.98, indicating that they may have good docking ability. ADH1C had the lowest binding energy when docked with Stigmasterol. CXCL8 had a slightly greater binding energy when docked with Quercetin, suggesting that Quercetin may not be as effective at binding CXCL8. CYP1A1, CYP1A2 CYP1A1, and CYP1A2 have higher binding energy with Kaempferol and Quercetin, indicating that CYP1A1 and CYP1A2 may be good targets for GGS. The above results are all computer analysis results and further experimental validation is still needed.

The above results suggest that GGS can interfere with the development of ALD by modulating targets related to fat metabolism and inflammatory response. The potential core targets include CYP1A1, CYP1A2, CXCL8, ADH1C, MMP1, SERPINE1, COL1A1, APOB, and MMP1. The main signaling pathways include the HIF-1 signaling pathway. This study elucidates to some extent the action mechanism of GGS in the treatment of ALD and is expected to guide the further application of GGS in the treatment of ALD.

The present study also has some limitations. The accuracy and comprehensiveness of the target and compound information originating from the database need to be improved. In practical application, whether different concoctions of single drugs in a Chinese herbal compound affect the drug effect and whether decoction produces other changes still need to be determined. These problems should be considered in future studies.

## Author contributions

**Conceptualization:** Jiakai Yang.

**Data curation:** Jiakai Yang, Ke Tang.

**Funding acquisition:** Qianqian Zhuang.

**Methodology:** Jiakai Yang, Xinli Liu.

**Project administration:** Xinli Liu.

**Software:** Jiakai Yang.

**Supervision:** Xinli Liu.

**Visualization:** Jiakai Yang.

**Writing – original draft:** Jiakai Yang.

**Writing – review & editing:** Jiakai Yang, Xinli Liu.

## Supplementary Material









## References

[1] Fatty Liver and Alcoholic Liver Disease Group of the Chinese Medical Association, Fatty Liver Disease Expert Committee of the Chinese Medical Association. Guidelines for the prevention and treatment of alcoholic liver disease (2018 updated edition). J Pract Liver Dis. 2018;21:170–6.

[2] GBD 2016 Causes of Death Collaborators. Global, regional, and national age-sex specified mortality for 264 causes of death, 1980–2016: a systematic analysis for the global burden of disease study 2016. Lancet. 2017;390:1151–210. 28919116 10.1016/S0140-6736(17)32152-9 PMC5605883

[3] Poynard T Bedossa P Opolon P. Natural history of liver fibrosis progression in patients with chronic hepatitis C. The OBSVIRC, METaViR, CLINIVIR, and DOSVIRC groups. Lancet. 1997;349:825–32. 9121257 10.1016/s0140-6736(96)07642-8

[4] World Health Organization. Global Status Report on Alcohol and Health 2018. Geneva, Switzerland: WHO; 2018.

[5] Gao X Liu L. Research progress in epidemiology and pathogenesis of alcoholic liver disease. Chin J Gastroenterol Imaging. 2016;6:62–5.

[6] Wu Y Li YR Yang ZB. Current status of research on the pathogenesis of alcoholic liver disease. J Clin Hepatobiliary Dis. 2020;36:2822–5.

[7] Pi JT Wang C Zhang JM. Epidemiological survey on alcohol consumption and alcoholic liver disease among permanent residents in Tongzhou District, Beijing. J Chronic Dis. 2022;23:712–6.

[8] Jin M Ande A Kumar A Kumar S. Regulation of cytochrome P450 2e1 expression by ethanol: role of oxidative stress-mediated pkc/jnk/sp1 pathway. Cell Death Dis. 2013;4:e554–e554. 23519123 10.1038/cddis.2013.78 PMC3615729

[9] Meng WW Liu HR Zhang WW. Research progress on the mechanism of action of traditional Chinese medicine against alcoholic liver disease. Chin Herbal Med. 2022;53:868–81.

[10] Portari GV Ovidio PP Deminice R Jordão AA. Protective effect of treatment with thiamine or benfotiamine on liver oxidative damage in rat model of acute ethanol intoxication. Life Sci. 2016;162:21–4. 27545821 10.1016/j.lfs.2016.08.017

[11] Szabo G Lippai D. Converging actions of alcohol on liver and brain immune signaling. Int Rev Neurobiol. 2014;118:359–80. 25175869 10.1016/B978-0-12-801284-0.00011-7

[12] Zhou T Luo Z Wu J Yang R Xiong C Li J. Study on the mechanism of action of Pueraria lobata in the treatment of alcoholic liver disease. Asia Pac Tradit Med. 2017;13:7–9.

[13] Liu S Ge L. Effect of Pueraria lobata on liver function injury in rats with alcoholic fatty liver. Hebei TCM. 2013;35:1703–4 + 1708.

[14] Tian W Wang W Han J Wang Q Yang Z. Effects of three anti-drinking prescriptions including Ge Gen San on Caspase3/8 activity in mice with acute alcohol intoxication. Chin Patent Med. 2012;34:2233–6.

[15] Tang D Yang Z Long F. Effect of Pueraria lobata on the expression of ICAM-1 in the liver microenvironment of colorectal cancer mice model. Shi-Zhen Guomao Guomao. 2012;23:2213–4.

[16] Dongxin T Zhu Y Fengxi L. Inhibitory effect of Pueraria lobata on adenocarcinoma of the colon in mice. J Med Res. 2012;41:79–81.

[17] Hopkins AL. Network pharmacology: the next paradigm in drug discovery. Nat Chem Biol. 2008;4:682–90. 18936753 10.1038/nchembio.118

[18] Hopkins AL. Network pharmacology. Nat Biotechnol. 2007;25:1110–1. 17921993 10.1038/nbt1007-1110

[19] Chen TA Chen AT Chen F. Application of computer simulation-based network pharmacology in modern Chinese medicine research. Biochemistry. 2022;8:159–62.

[20] Jinlong R Peng L Jinan W. TCMSP: a database of systems pharmacology for drug discovery from herbal medicines. J Cheminform. 2014;6:13. 24735618 10.1186/1758-2946-6-13 PMC4001360

[21] Liu Z Guo F Wang Y. BATMAN-TCM: a Bioinformatics Analysis Tool for Molecular mechANism of Traditional Chinese Medicine. Sci Rep. 2016;6:21146. 26879404 10.1038/srep21146 PMC4754750

[22] Ji ZL Zhou H Wang JF Han LY Zheng CJ Chen YZ. Traditional Chinese medicine information database. J Ethnopharmacol. 2006;103:501. 16376038 10.1016/j.jep.2005.11.003

[23] Piñero J Bravo A Queralt-Rosinach N. DisGeNET: a comprehensive platform integrating information on human disease-associated genes and variants. Nucleic Acids Res. 2017;45:D833–9. 27924018 10.1093/nar/gkw943 PMC5210640

[24] Piñero J Ramírez-Anguita JM Saüch-Pitarch J. The DisGeNET knowledge platform for disease genomics: 2019 update. Nucleic Acids Res. 2020;48:D845–55. 31680165 10.1093/nar/gkz1021 PMC7145631

[25] Safran M Rosen N Twik M. The GeneCards suite chapter. In: Practical Guide to Life Science Databases. https://www.genecards.org/; 2022:27–56.

[26] Otasek D Morris JH Bouças J Pico AR Demchak B. Cytoscape automation: empowering workflow-based network analysis. Genome Biol. 2019;20:185. 31477170 10.1186/s13059-019-1758-4 PMC6717989

[27] Szklarczyk D Morris JH Cook H. The STRING database in 2017: quality-controlled protein–protein association networks, made broadly accessible. Nucleic Acids Res. 2017;45:D362–8. 27924014 10.1093/nar/gkw937 PMC5210637

[28] Barrett T Wilhite SE Ledoux P. NCBI GEO: archive for functional genomics data sets—update. Nucleic Acids Res. 2013;41:D991–5. 23193258 10.1093/nar/gks1193 PMC3531084

[29] Sherman BT Hao M Qiu J. DAVID: a web server for functional enrichment analysis and functional annotation of gene lists (2021 update). Nucleic Acids Res. 2022;50:W216–21. 35325185 10.1093/nar/gkac194 PMC9252805

[30] Huang DW Sherman BT Lempicki RA. Systematic and integrative analysis of large gene lists using DAVID bioinformatics resources. Nat Protoc. 2009;4:44–57. 19131956 10.1038/nprot.2008.211

[31] Jumper J Evans R Pritzel A. Highly accurate protein structure prediction with AlphaFold. Nature. 2021;596:583–7. 34265844 10.1038/s41586-021-03819-2 PMC8371605

[32] Varadi M Anyango S Deshpande M. AlphaFold protein structure database: massively expanding the structural coverage of protein-sequence space with high-accuracy models. Nucleic Acids Res. 2021;50:D439–44. 10.1093/nar/gkab1061 PMC8728224 34791371

[33] PDBe-KB consortium. PDBe-KB: collaboratively defining the biological context of structural data. Nucleic Acids Res. 2022;50:534–42. 10.1093/nar/gkab988 PMC8728252 34755867

[34] Kim S Chen J Cheng T. PubChem in 2021: new data content and improved web interfaces. Nucleic Acids Res. 2021;49:D1388–95. 33151290 10.1093/nar/gkaa971 PMC7778930

[35] Goodsell DS Olson AJ. Automated docking of substrates to proteins by simulated annealing. Proteins. 1990;8:195–202. 2281083 10.1002/prot.340080302

[36] Adasme MF Linnemann KL Bolz SN. PLIP 2021: expanding the scope of the protein–ligand interaction profiler to DNA and RNA. Nucleic Acids Res. 2021;49:W530–4. 33950214 10.1093/nar/gkab294 PMC8262720

[37] The PyMOL Molecular Graphics System, Version 2.0. Schrödinger, LLC.

[38] Laskowski RA Swindells MB. LigPlot+: multiple ligand–protein interaction diagrams for drug discovery. J Chem Inf Model. 2011;51:2778–86. 21919503 10.1021/ci200227u

[39] Jiménez J Doerr S Martínez-Rosell G Rose AS De Fabritiis G. DeepSite: protein binding site predictor using 3D-convolutional neural networks. Bioinformatics. 2017;33:3036–42. 28575181 10.1093/bioinformatics/btx350

[40] Heatmap was plotted by https://www.bioinformatics.com.cn (last accessed on October 31, 2022), an online platform for data analysis and visualization.

[41] Gillespie M Jassal B Stephan R. The reactome pathway knowledgebase 2022. Nucleic Acids Res. 2021;50:gkab1028. 10.1093/nar/gkab1028 PMC8689983 34788843

[42] Tan J Mckenzie C Potamitis M Thorburn AN Mackay CR Macia L. The role of short-chain fatty acids in health and disease. Adv Immunol. 2014;121:91–119. 24388214 10.1016/B978-0-12-800100-4.00003-9

[43] Bondy SC. Ethanol toxicity and oxidative stress. Toxicol Lett. 1992;63:231–41. 1488774 10.1016/0378-4274(92)90086-y

[44] Bailey SM Cunningham CC. Contribution of mitochondria to oxidative stress associated with alcoholic liver disease. Free Radic Biol Med. 2002;32:11–6. 11755312 10.1016/s0891-5849(01)00769-9

[45] Crabb DW Galli A Fischer M You M. Molecular mechanisms of alcoholic fatty liver: role of peroxisome proliferator-activated receptor alpha. Alcohol. 2004;34:35–8. 15670663 10.1016/j.alcohol.2004.07.005

[46] You M Fischer M Deeg MA Crabb DW. Ethanol induces fatty acid synthesis pathways by activation of sterol regulatory element-binding protein (SREBP). J Biol Chem. 2002;277:29342–7. 12036955 10.1074/jbc.M202411200

[47] Tomita K Tamiya G Ando S. AICAR, an AMPK activator, has protective effects on alcohol-induced fatty liver in rats. Alcohol Clin Exp Res. 2005;29:240S–5S. 16385230 10.1097/01.alc.0000191126.11479.69

[48] Lieber CS. Cytochrome P-4502E1: its physiological and pathological role. Physiol Rev. 1997;77:517–44. 9114822 10.1152/physrev.1997.77.2.517

[49] Hansson T Tindberg N Ingelman-Sundberg M Köhler C. Regional distribution of ethanol-inducible cytochrome P450 IIE1 in the rat central nervous system. Neuroscience. 1990;34:451–63. 2333153 10.1016/0306-4522(90)90154-v

[50] Shi Z Zhang C Lei H. Structural Insights into amelioration effects of quercetin and its glycoside derivatives on NAFLD in mice by modulating the gut microbiota and host metabolism. J Agric Food Chem. 2022;70:14732–43. 36351282 10.1021/acs.jafc.2c06212

[51] Zhao X Wang C Dai S. Quercetin protects ethanol-induced hepatocyte pyroptosis via scavenging mitochondrial ROS and Promoting PGC-1α-regulated mitochondrial homeostasis in L02 cells. Oxid Med Cell Longevity. 2022;2022:1–15. 10.1155/2022/4591134 PMC9308520 35879991

[52] Inokuchi S Tsukamoto H Park E Liu Z-X Brenner DA Seki E. Toll-like receptor 4 mediates alcohol-induced steatohepatitis through bone marrow-derived and endogenous liver cells in mice. Alcohol Clin Exp Res. 2011;35:1509–18. 21463341 10.1111/j.1530-0277.2011.01487.x PMC3131439

[53] Chen L Xia S Wang S. Naringenin is a potential immunomodulator for inhibiting liver fibrosis by inhibiting the cGAS-STING pathway. J Clin Transl Hepatol. 2023;11:26–37. 36406329 10.14218/JCTH.2022.00120 PMC9647116

[54] Cai X Wang S Wang H. Naringenin inhibits lipid accumulation by activating the AMPK pathway in vivo and vitro. Food Sci Human Wellness. 2023;12:1174–83.

[55] Anania FA Womack L Potter JJ Mezey E. Acetaldehyde enhances murine alpha2(I) collagen promoter activity by Ca2+-independent protein kinase C activation in cultured rat hepatic stellate cells. Alcohol Clin Exp Res. 1999;23:279–84. 10069557

