# Supplementary figures and images for: Exploring the action mechanism of Gegensan in the treatment of alcoholic liver disease based on network pharmacology and bioinformatics
Source: Medicine (Baltimore). 2024 Jun 21;103(25):e38315. doi: 10.1097/MD.0000000000038315 (PMC11191986; doi:10.1097/MD.0000000000038315)

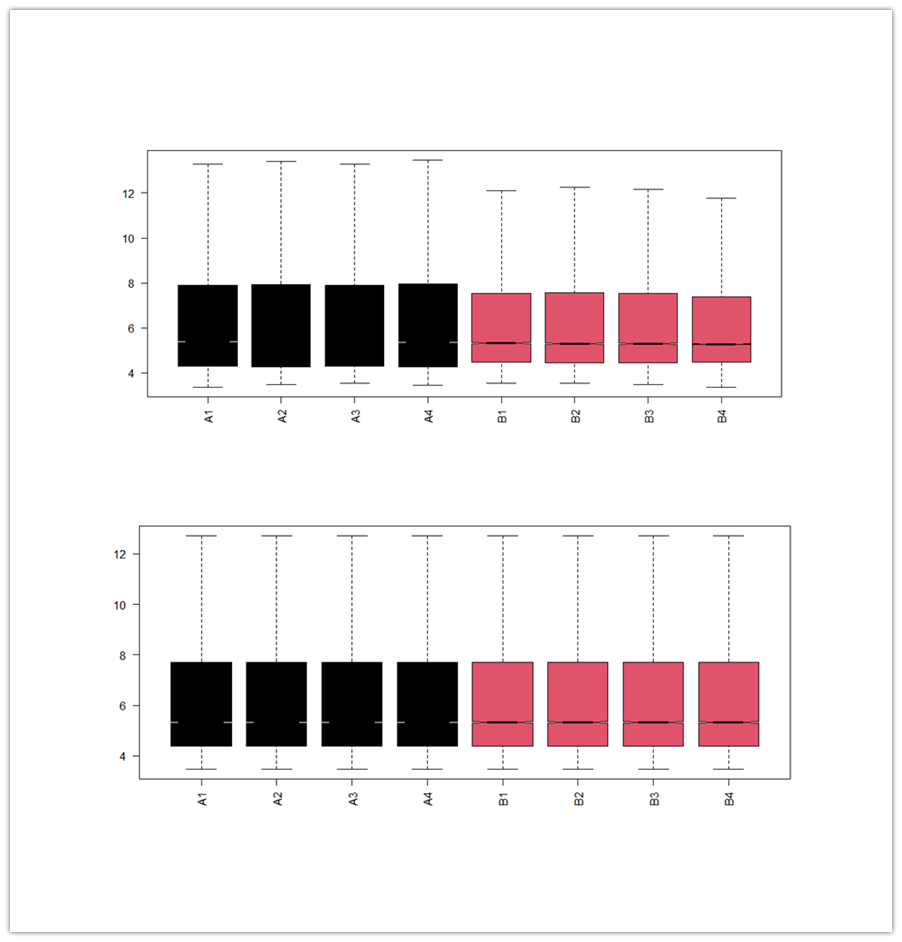


Supplementary figure 1. Box plot before and after normalization of GSE100901 data

Supplement: Supplementary file 1 [file medi-103-e38315-s001.docx]
