# Supplementary material for: Exploring the action mechanism of Gegensan in the treatment of alcoholic liver disease based on network pharmacology and bioinformatics
Source: Medicine (Baltimore). 2024 Jun 21;103(25):e38315. doi: 10.1097/MD.0000000000038315 (PMC11191986; doi:10.1097/MD.0000000000038315)
Supplement: Supplementary file 2 [file medi-103-e38315-s002.docx]

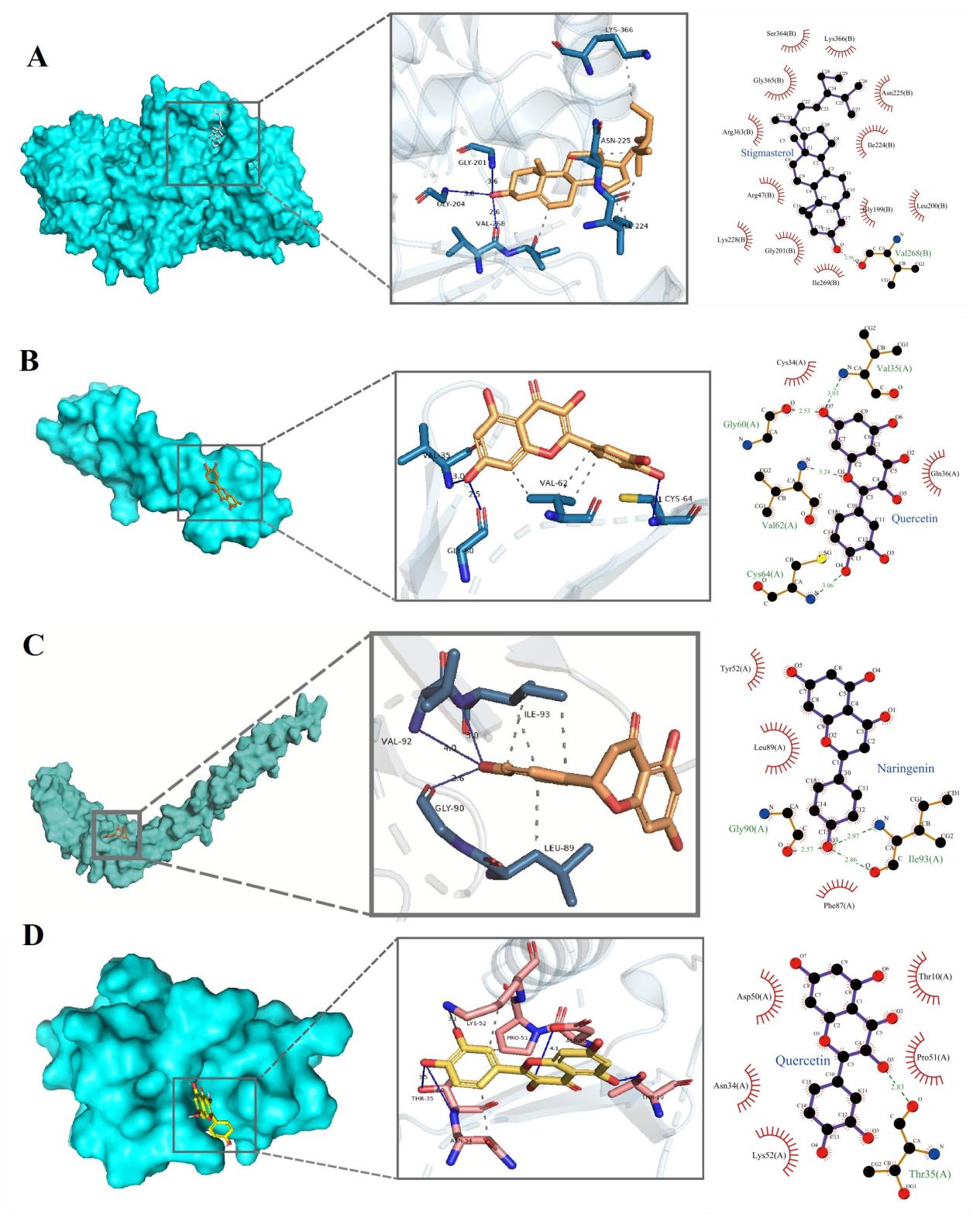


Supplementary figure 2. Binding conformations of GGS-ALD core targets to active ingredient docking compounds. (A) ADH1C and Stigmasterol.(B) COL1A1 and Quercetin.(C) APOB and Naringenin.(D) CXCL8 and Quercetin.(E) CYP1A1 and Quercetin.(F) CYP1A1 and Kaempferol.(G) CYP1A2 and Quercetin.(H) CYP1A2 and Kaempferol.(I) MMP1 and Kaempferol. (H) CYP1A2 and Kaempferol.(I) MMP1 and Kaempferol.(J) MMP1 and Quercetin.(K) SERPINE1 and Quercetin.


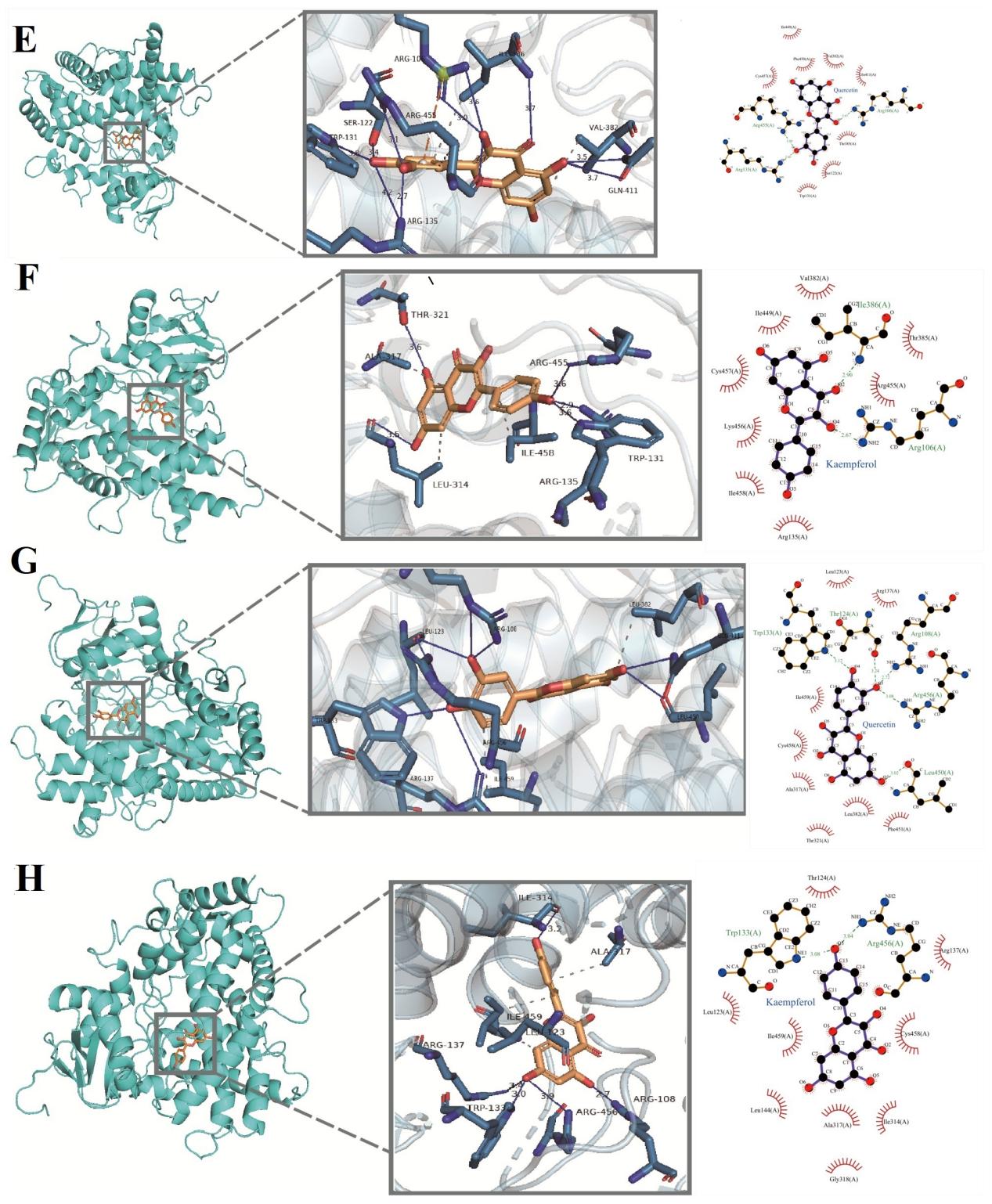


Supplementary figure 2. (continued).


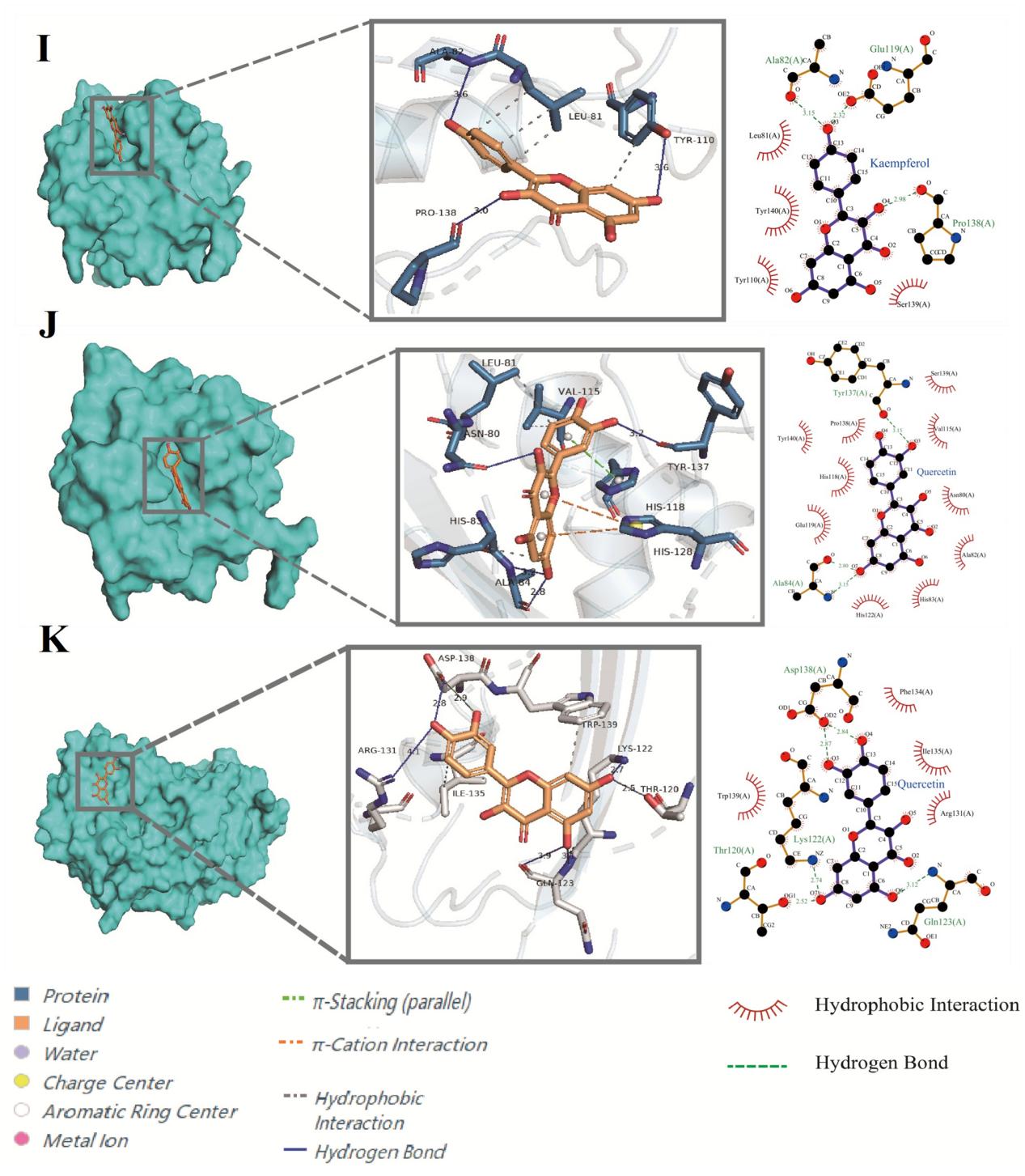


Supplementary figure 2. (continued).
