# Supplementary material for: Exploring the action mechanism of Gegensan in the treatment of alcoholic liver disease based on network pharmacology and bioinformatics
Source: Medicine (Baltimore). 2024 Jun 21;103(25):e38315. doi: 10.1097/MD.0000000000038315 (PMC11191986; doi:10.1097/MD.0000000000038315)
Supplement: Supplementary file 3 [file medi-103-e38315-s003.docx]

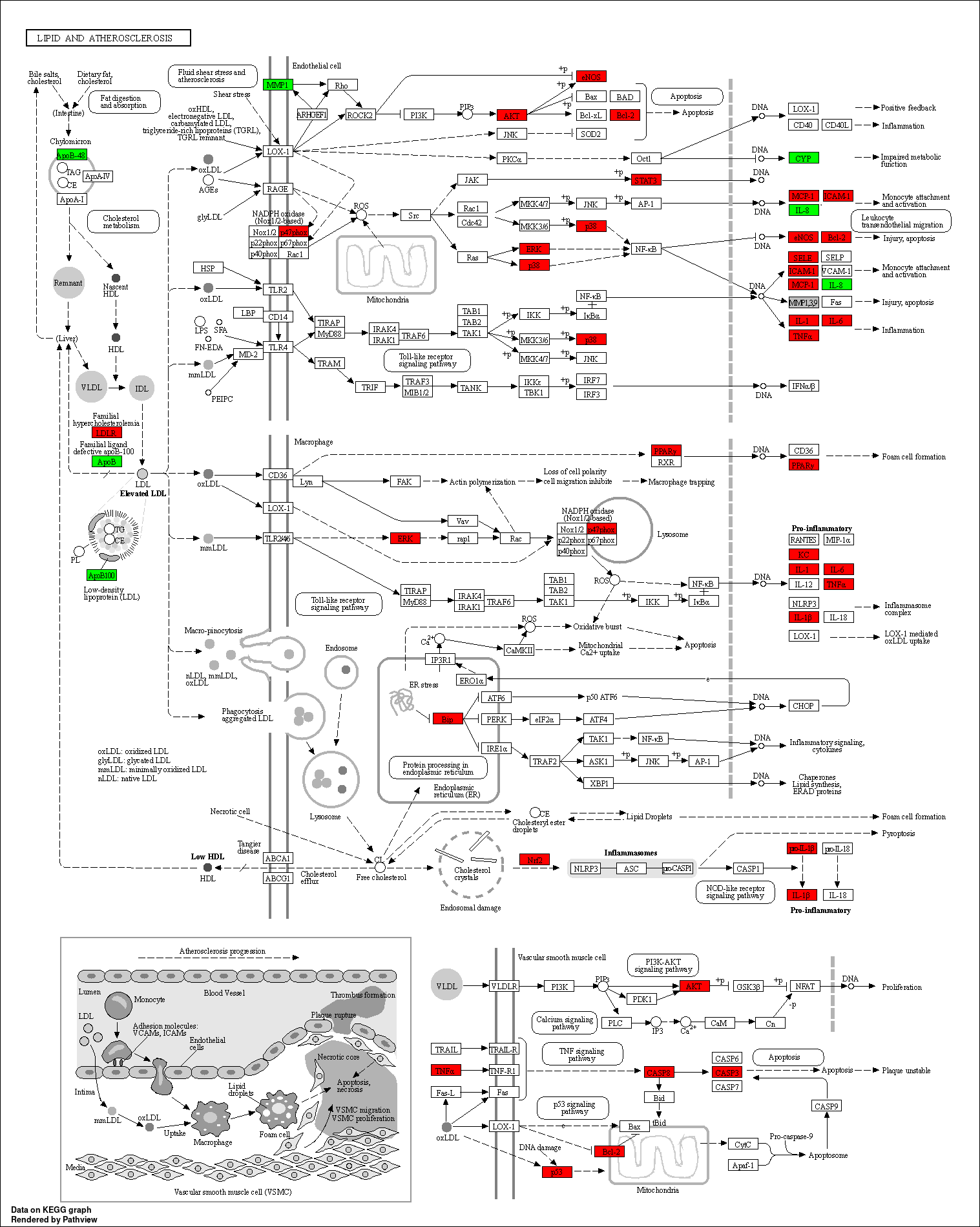


Supplementary figure 3. Map of lipid and atherosclerosis pathways. Core targets are filled in green and common ALD-GGS targets are filled in red.
